# Tumor-Stroma Crosstalk in Bone Tissue: The Osteoclastogenic Potential of a Breast Cancer Cell Line in a Co-Culture System and the Role of EGFR Inhibition

**DOI:** 10.3390/ijms18081655

**Published:** 2017-07-29

**Authors:** Laura Mercatali, Federico La Manna, Giacomo Miserocchi, Chiara Liverani, Alessandro De Vita, Chiara Spadazzi, Alberto Bongiovanni, Federica Recine, Dino Amadori, Martina Ghetti, Toni Ibrahim

**Affiliations:** 1 Osteoncology and Rare Tumors Center, Istituto Scientifico Romagnolo per lo Studio e la Cura dei Tumori (IRST) IRCCS, 47014 Meldola, Italy; federico.lamanna@lumc.nl (F.L.M) giacomo.miserocchi@irst.emr.it (G.M.); chiara.liverani@irst.emr.it (C.L.); alessandro.devita@irst.emr.it (A.D.V.); chiara.spadazzi@irst.emr.it (C.S.); alberto.bongiovanni@irst.emr.it (A.B.); federica.recine@irst.emr.it (F.R.); editing@irst.emr.it (D.A.); martina.ghetti@irst.emr.it (M.G.); toni.ibrahim@irst.emr.it (T.I.); 2 Biomedical and Neuromotor Sciences Department, University of Bologna, 40123 Bologna, Italy

**Keywords:** breast cancer, co-culture, osteoclasts, mesenchymal stromal cells, non-canonical osteoclastogenesis

## Abstract

Although bone metastases represent a major challenge in the natural history of breast cancer (BC), the complex interactions involved have hindered the development of robust in vitro models. The aim of this work is the development of a preclinical model of cancer and bone stromal cells to mimic the bone microenvironment. We studied the effects on osteoclastogenesis of BC cells and Mesenchymal stem cells (MSC) cultured alone or in combination. We also analyzed: (a) whether the blockade of the Epithelial Growth Factor Receptor (EGFR) pathway modified their influence on monocytes towards differentiation, and (b) the efficacy of bone-targeted therapy on osteoclasts. We evaluated the osteoclastogenesis modulation of human peripheral blood monocytes (PBMC) indirectly induced by the conditioned medium (CM) of the human BC cell line SCP2, cultured singly or with MSC. Osteoclastogenesis was evaluated by TRAP analysis. The effect of the EGFR blockade was assessed by treating the cells with gefitinib, and analyzed with the 3-(4,5-dimethylthiazol-2-yl)-2,5-diphenyltetrazolium bromide (MTT) assay and Western Blot (WB). We observed that SCP2 co-cultured with MSC increased the differentiation of PBMC. This effect was underpinned upon pre-treatment of the co-culture with gefitinib. Co-culture of SCP2 with MSC increased the expression of both the bone-related marker Receptor Activator of Nuclear Factor κB (RANK) and EGFR in BC cells. These upregulations were not affected by the EGFR blockade. The effects of the CM obtained by the cells treated with gefitinib in combination with the treatment of the preosteoclasts with the bone-targeted agents and everolimus enhanced the inhibition of the osteoclastogenesis. Finally, we developed a fully human co-culture system of BC cells and bone progenitor cells. We observed that the interaction of MSC with cancer cells induced in the latter molecular changes and a higher power of inducing osteoclastogenesis. We found that blocking EGFR signaling could be an efficacious strategy for breaking the interactions between cancer and bone cells in order to inhibit bone metastasis.

## 1. Introduction

Bone metastases are a common event in breast cancer (BC) patients [1], often leading to severe symptoms such as pain, bone fractures, spinal cord compression and hypercalcemia [2]. The mechanisms underlying BC-derived bone metastases have been intensively investigated but the complex interactions involved have hindered both the development of comprehensive in vitro systems [3] and the generation of translational benefits [4,5].

Mesenchymal stromal cells (MSC) are multipotent stem cells precursors of tissue-specific cell lineages in many adult tissues [6]. Within the bone marrow, MSC give rise to both bone stromal cells, i.e., osteoblasts, osteocytes, adipocytes, and more specialized, niche-maintaining cells such as CAR cells, leptin-receptor-positive cells, and sinusoid-associated pericytes [7]. Together with osteoblasts, MSC express two essential factors for the maturation of osteoclasts, the giant, multinucleated, monocyte-derived cells responsible for bone resorption: the Receptor Activator of Nuclear Factor kappa-Bligand (RANKL) and the macrophage colony-stimulating factor (M-CSF) [8]. The inhibition of either the maturation or the activity of osteoclasts has already proven effective in treating bone metastases [1,9], and has led to the development of many osteoclast-targeted drugs [10,11]. These pharmacological agents, which constitute the bone-targeted therapy, are mainly represented by the anti-RANKL antibody denosumab (Den), and bisphosphonates, such as zoledronic acid (Zol). Everolimus (Eve) has been recently considered suitable for targeting bone metastases as it inhibits both cancer cells and osteoclasts. [12,13,14]. EGFR, also known as ErbB-1 or HER1, is one of the four members of the ErbB tyrosine kinases receptors family. EGFR is frequently amplified or overexpressed in many neoplastic lesions, including lung, breast, colorectal, head and neck, pancreatic and gastric cancers [15]. A higher expression of EGFR in “triple-negative” BC has been shown to correlate with a higher incidence of metastases [16]. This receptor is also expressed by MSC, and the activation of the EGFR pathway is involved with MSC in the regulation of RANKL and OPG secretion [16]. Thus, a deregulated EGFR signaling in MSC may alter the homeostatic ratio of RANKL/OPG of the bone microenvironment, initiating a “vicious cycle” that leads to the uncoupling of the physiological balance between bone erosion and bone deposition, driving it towards cancer cell metabolism [8,16,17]. Gefitinib (Gef), a selective, reversible inhibitor of EGFR, effective in treating locally advanced or metastatic non-small-cells lung cancer (NSCLC), has received FDA approval as a first-line treatment in NSCLC patients [18]. In spite of the antitumor activity shown by Gef both in vitro and xenograft models of breast and prostate cancers [19,20,21,22], phase-I/-II clinical trials on advanced BC patients treated with Gef yielded conflicting results, with little assessed clinical benefits for patients [8,23,24,25,26,27]. However, treatment of bone metastases with Gef has also been explored [28,29], based on the unexpected observation that treatment with this type of tyrosine kinase inhibitor (TKI) could relieve bone pain in BC patients [30,31]. Ultimately, the available data from both preclinical studies and clinical observations may pave the way for the use of Gef and other TKIs for the treatment of bone metastases.

**Figure 1:**
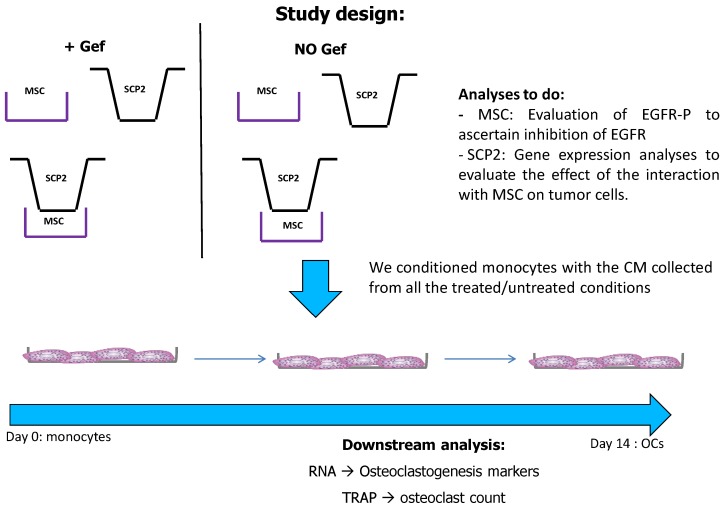
Experimental design: the preclinical model optimized in this paper includes a 2-phase cell culture; in the first phase cancer cells and Mesenchymal stem cells (MSC) were co-cultured sharing medium using transwell inserts. The media obtained from SCP2, MSC and the COCO were collected and used to condition the monocytes toward differentiation throughout the osteoclastogenic assay (CM changed every 2–3 days; assay total duration, 14 days). The conditioning of the monocyte with the CM derived from all the samples during the second phase of the experiment (indirect COCO).

The aim of this study was to develop a preclinical model for studying the effects of the crosstalk between stromal cells and cancer cells on osteoclastogenesis. Furthermore, we investigated if the blockade of the EGFR pathway in cancer and bone cells could have an effect on the bone microenvironment. We combined direct sharing medium and indirect co-cultures (COCOs) (Figure 1) in order to understand the molecular relation between cancer cells and MSC and their effect on osteoclasts. Firstly, we co-cultured cancer cells and MSC by direct sharing medium COCO. We then performed osteoclastogenesis assays in presence of the CM obtained from the cells previously mono- and co-cultured. These interactions were then challenged by treating the COCOs of stromal and cancer cells with Gef.

## 2. Results

### 2.1. Mesenchymal stem cells (MSC) Induce the Expression of RANK and EGFR in Cancer Cells

**Figure 2:**
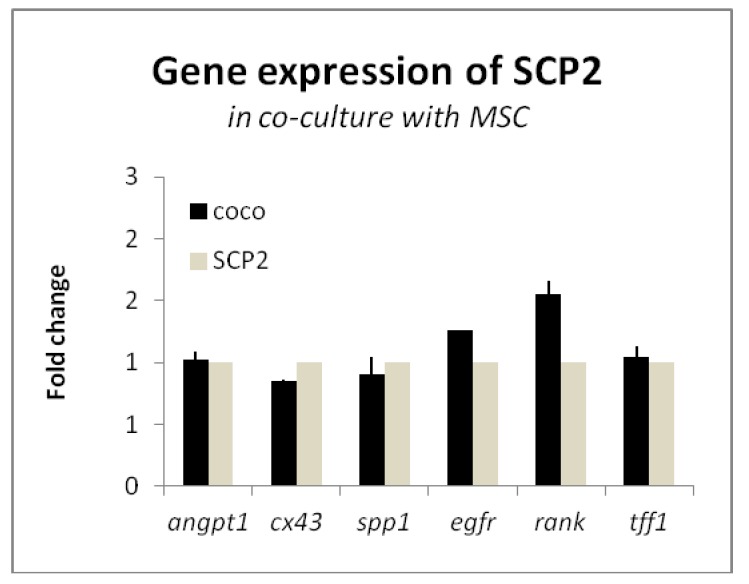
Co-culture with MSC upregulates EGFR and RANK expression in SCP2. Expression of *angpt1*, *cx43*, *spp1*, *egfr*, *rank* and *tff1* in SCP2 cells co-cultured with MSC. Fold change compared to SCP2 monoculture at baseline.

We first investigated the effects of the COCO with MSC on the SCP2 gene expression in order to understand if cancer cells behave differently when in contact with bone microenvironment factors. We selected markers recapitulating different hallmarks of cancer progression, in particular bone metastasis. We selected 3 markers of osteomimicry: connexin 43 (cx43), osteopontin (spp1), and RANK. Osteomimicry is the capability of cancer cells to colonize the bone in order to form metastasis, and express the genes, that are usually expressed by bone cells, by adapting to the new microenvironment [32,33]. Marker cx43 is usually expressed on osteoblasts and osteocytes, and it is in charge of mechanotransduction and the control of body mass. Marker spp1 is a matrix protein involved in the attachment of osteoclasts to the bone matrix [34] RANK is a receptor of osteoclast precursors that, when binding to the RANK ligand, mediates their differentiation into mature osteoclasts. It has been shown that BC cells with high tropism to the bone often overexpress RANK [35,36]. We assayed the expression of TFF1, a marker expressed at higher levels in primary BC patients with relapse to the bone [37] and angiopoietin-1 (angpt1), which is involved in the angiogenic response of the osteomimicry-related markers [38,39]. We added EGFR to the panel, as one of the aims of this study was to understand the role of the EGFR pathway in the bone microenvironment. To this aim, the expression of markers related to cell–cell communication, invasion and bone marrow colonization was analyzed in SCP2 cells after co-culturing for 72 h with MSC. SCP2 cells were cultured with MSC, using the inserts cultured with cancer cells that had been laid on well plates where MSC had been previously seeded. This type of COCO allows for media sharing and interactions between cells seeded on different floors (Figure 1). Data on cancer cell gene expression were normalized using baseline SCP2 culture as reference. As shown in Figure 2, we found that the COCO with MSC showed a trend towards the upregulation of two genes involved in the bone vicious cycle: EGFR and RANK [36,40,41], the latter showing an increase by over 1.5 times the basal level.

### 2.2. Cancer Cells and MSC Contribute to Osteoclastogenesis

In order to understand if cancer cells and MSC affect bone microenvironment contributing to osteoclastogenesis, we dissected the contribution of either SCP2 or MSC on osteoclastogenesis, by supplementing pre-osteoclasts with the CM of either the SCP2 or the MSC mono-cultures and the SCP2-MSC COCO. To achieve the COCO CM, we harvested the CM both after 24 h (Early-CM) and after 72 h (Late-CM) of COCO. In order to consider the osteoclastogenic power of CM with respect to the positive and negative control, we measured the average number of TRAP-positive osteoclast cell-like cells and their average surface area, given that a large surface area is one of the features of mature osteoclasts.

#### Cancer cells and COCO promote Osteoclastogenesis

**Figure 3:**
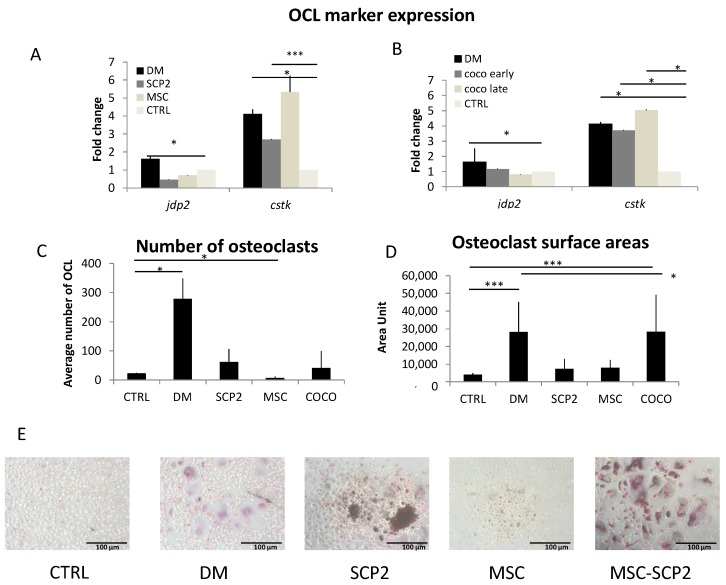
SCP2 and MSC mono-culture and COCO induce osteoclastogenesis. Expression of *jdp2* and *ctsk* in osteoclasts cultured either in DM or in pre-osteoclast medium supplemented with CM from: (**A**) SCP2 or MSC monoculture; (**B**) SCP2-MSC COCO after 24 h (Early-CM) or 72 h (Late-CM) of COCO. qPCR data refer to RNA from pre-osteoclasts. Data were normalized on pre-osteoclasts. *t*-test was performed comparing the gene expression of the different conditions with the control. * *p* < 0.05; *** *p* < 0.01. Average number (**C**) and average surface area (**D**) of TRAP-positive osteoclasts induced by culture in DM, pre-osteoclast medium (CTRL) or pre-osteoclast medium supplemented with SCP2-CM (SCP2), with MSC-CM (MSC) or with CM from SCP2-MSC COCO; *t*-test was performed for all conditions taking the negative control as control (CTRL). * *p* < 0.05; *** *p* < 0.01; (**E**) pictures of osteoclasts on day 14 obtained in the different experimental conditions performed at 10× magnification.

At a molecular level, both CM from SCP2 and MSC induced osteoclastogenesis upregulating the osteoclast marker cathepsin k (*ctsk*), as shown in Figure 3A,B, with MSC-derived CM. We observed no upregulation of the early osteoclast-specific transcription factor Jun dimerization protein 2 (*jdp2*), probably because it had been already turned off on day 14 of differentiation [42]. Compared to undifferentiated pre-osteoclasts (negative control, i.e., monocytes cultured without GF supplementation), SCP2-derived CM showed a trend towards the induction of osteoclastogenesis, both as for the number of TRAP-positive osteoclasts (Figure 3C) and the surface area measurement (Figure 3D).

The CM from COCO induced a significant increase of the expression of *ctsk* in pre-osteoclast cultures, without affecting the expression of *jdp2*, and with no major differences between Early- and Late-CM (Figure 3B). Based on this result, we performed the TRAP assay on the osteoclasts cultured with Late-CM only (COCO), comparing data with both the positive and negative controls. The average surface area of the osteoclasts generated by the CM derived from the SCP2-MSC COCO was comparable to that of the osteoclasts cultured in the positive control, and was significantly higher than the negative control. It however did not affect the average number of osteoclasts (Figure 3C,D).

Osteoclasts are not the only TRAP-positive polinucleated cells of a monocytic origin. In a previous work we confirmed that the osteoclast cell-like cells positive to TRAP are real osteoclasts, as we observed the presence of two specific osteoclast markers, i.e., Actin ring and calcitonin receptor (CTR), both in the negative control and in the differentiation medium (DM) condition [12].

### 2.3. Gef Induces RANK and EGFR mRNA Expression and Inhibits the EGFR Pathway at the Protein Level

**Figure 4:**
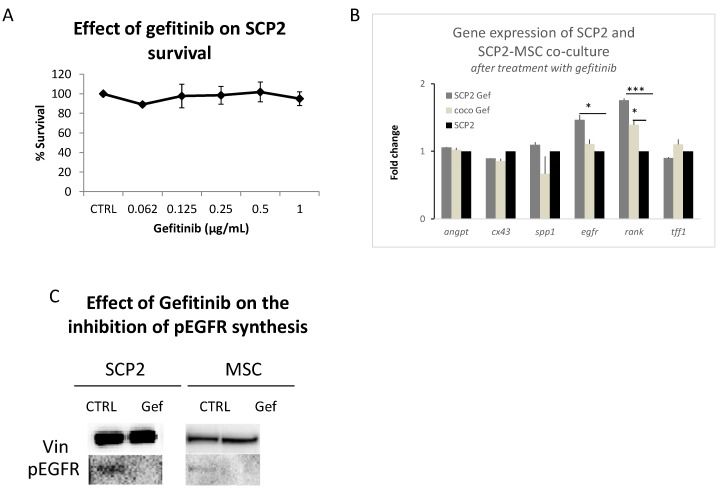
Gef inhibits the EGFR pathway and modulates cancer cell gene expression analyses. (**A**) Survival rate of SCP2 treated with Gef. Data were normalized to untreated sample (CTRL); (**B**) Expression of *angpt1*, *cx43*, *spp1*, *egfr*, *rank* and *tff1* in SCP2 cells (SCP2 Gef) or in SCP2-MSC COCO (TRW Gef) treated with Gef 1µg/mL. Fold change compared to SCP2 culture at baseline. * *p* < 0.05. *** *p* < 0.01; (**C**) Synthesis of phosphorylated-EGFR (pEGFR) in SCP2 and MSC in absence (CTRL) and treated with Gef. In each condition we added EGFR with an incubation step of 10 min (50 ng/mL).

To investigate the involvement of the EGFR pathway on the crosstalk of SCP2 cells and MSC, we treated both SCP2 and MSC monocultures and COCOs with Gef. As the first step, we tested the effect of different concentrations of Gef on SCP2 cells. As expected, the SCP2 cell line, like the parental cell line MDA-MB-231 [19], was not very sensitive to the EGFR blockade induced by Gef (Figure 4A). We then analyzed the expression of *angpt1*, *cx43*, *spp1*, *egfr*, *rank* and *tff1* after treating the SCP2 culture or the SCP2-MSC COCO with Gef at 1 µg/mL (plasmatic peak concentration) for 24 h (Figure 4B), after normalizing the gene expression on the SCP2 culture at baseline, to evaluate any molecular changes. Gef-treated cells showed a modulation only of RANK and EGFR compared to the negative control (monocytes cultured without GF and CM). RANK increased both in SCP2 cultured singly (Figure 3B) and in COCO. We observed the same trend also for EGFR (Figure 3). The treatment with Gef induced a significant upregulation of *rank*, compared to untreated baseline SCP2 cells, once again showing a gene expression profile similar to untreated SCP2-MSC COCO.

To confirm that our observations depended on the blocking of EGFR, we tested the activation of the EGFR pathway at the protein level, both in SCP2 and MSC cultures. After the addition of EGF cytokine we detected the activation of the EGFR pathway, in terms of phospho-EGFR, in the cultures of SCP2 and MSC Gef treatment inhibited EGFR signaling, as we observed lack of phospho-EGFR in the treated cells (Figure 4C).

### 2.4. Gefitinib Impairs Osteoclastogenesis Induced by MSC-SCP2 COCO

**Figure 5:**
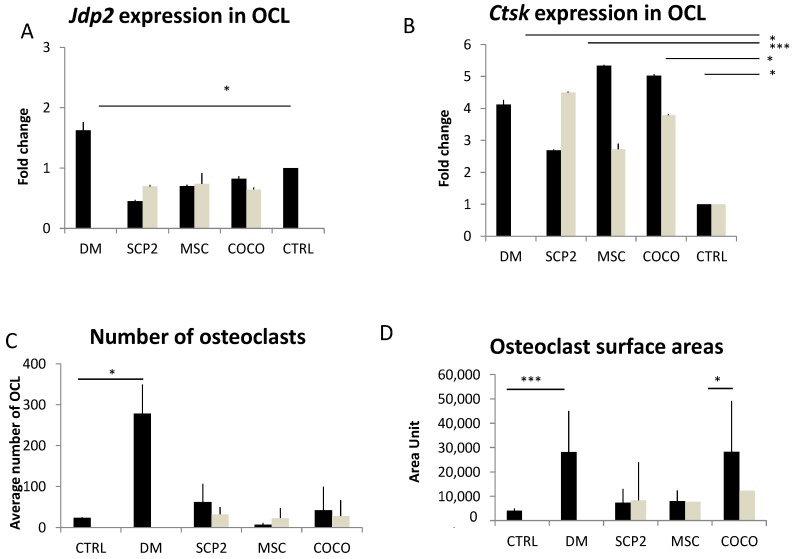
Gef impairs osteoclastogenesis induced by MSC-SCP2 COCO. Expression of *jdp2* (**A**) and *ctsk* (**B**) in osteoclasts cultured either in DM or with CM from SCP2, MSC or SCP2-MSC COCO previously treated (grey bars) or not (black bars) with Gef 1 µg/ml. Significance is compared to undifferentiated pre-osteoclasts (CTRL); Average number (**C**) and average surface area (**D**) of osteoclasts cultured in pre-osteoclast medium (CTRL), in DM or with CM from SCP2, MSC or SCP2-MSC COCO previously treated (grey bars) or not (black bars) with Gef 1µg/mL. * *p* < 0.05; *** *p* < 0.01. Gef was not added to CTRL and DM conditions in this context (absence of grey bars).

We investigated the effect of *Gef* on the osteoclastogenic potential of CM from SCP2 and MSC mono- and co-cultures. We observed a statistically significant downregulation of *ctsk* in the CM from Gef-treated MSC and COCO (Figure 4A,B). The treatment did not inhibit the osteoclastogenic power of the CM obtained from SCP2. This could mean that the osteoclastogenic induction by MSC depends on the EGFR pathway; this trend was confirmed by the data obtained from counting the number of osteoclasts and quantifying the surface area, with statistical significance reached by the decreased mean surface area Figure 5C,D. No significant modulation of *jdp2* could be detected. At the cytological level, the CM of the *Gef*-treated cultures reduced the osteoclastogenic potential as shown in Figure 4B. We observed no statistically different level of osteoclastogenesis from the negative control, neither in the number (Figure 5C) nor in the surface area (Figure 5D) of osteoclasts.

### 2.5. Bone-Targeted Therapy Empower Gef Inhibition of Osteoclastogenesis

**Figure 6:**
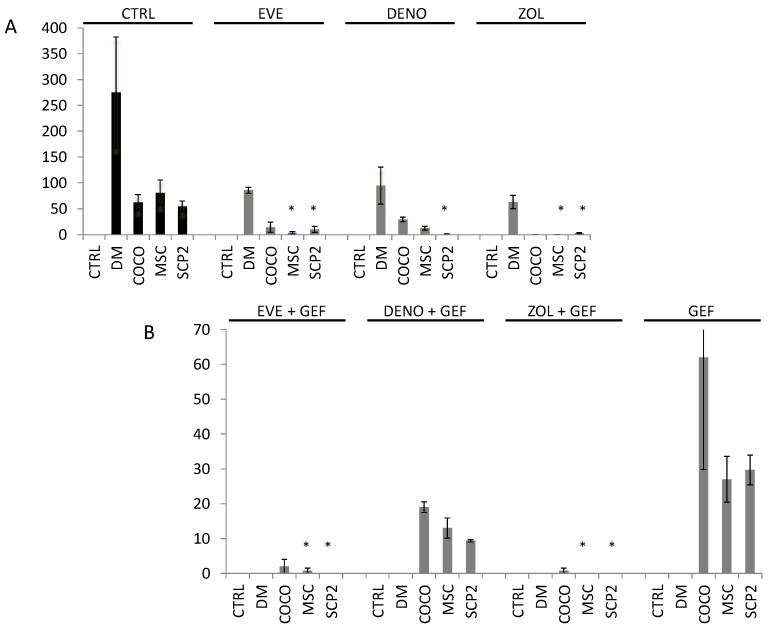
Eve and Zol on osteoclasts together with Gef treatment on MSC and SCP2 totally abrogated osteoclastogenesis. (**A**) Osteoclastogenesis assay from PBMC treated with bone-targeted therapy drugs (Den and Zol) and Eve. Osteoclastogenesis differentiation was evaluated in PBMC cultured with basal media with the addition of growth factors MCSF and RANKL, or with CM from MSC-SCP2 in COCO or mono-culture and treated with drugs; (**B**) Drug effects on osteoclastogenesis in combination with Gef. During the osteoclastogenic assay bone-targeted therapy drugs and Eve were added. Significance was compared to untreated osteoclasts; * *p* < 0.05.

As in clinical practice bone metastasis patients are usually treated with bone target therapy as Den and Zol, we treated the monocytes with these drugs to understand the impact of the inhibition of EGFR on standard treatment. We also tested the effect of Eve, as we previously had observed its strong effect on osteocolstogenesis inhibition. We employed CM from MSC, SCP2 and SCP2-MSC COCO for each treatment. At the end of the assay, the number of osteoclasts was considerably decreased in all the conditions. Compared to the control without treatment, all the drugs showed an important inhibitory effect on the percentage of differentiated osteoclasts, both in DM and CM conditions. Zol had the strongest effects on this reduction. We also evaluated the effects of the CM derived from the cells treated with Gef. All the combinations underpinned the development of mature osteoclasts (Figure 6B). The greatest effect on osteoclast development was performed by Zol, which showed a complete inhibition of the CM from MSC and SCP2 conditions (Figure 6B).

## 3. Discussion

This study aimed to evaluate the crosstalk between BC cells and bone-derived stromal cells through the development of a fully human preclinical model useful for studying drug activity on the bone microenvironment. We used the SCP2 BC cell line, an MDA-MB-231-derived triple-negative cell line with an intrinsic bone metastatic signature [43], which constitutes a good candidate for investigating BC metastasis in vitro.

Several studies have investigated the interactions of BC cells with primary osteoblasts or with osteogenically differentiated [19,20,21,22,44] and undifferentiated [45,46] MSC.Since undifferentiated MSC represent a key mediator in the bone microenvironment crosstalk [44,47,48], we investigated their interactions with cancer cells. First, we allowed the MSC to interact through medium sharing with the SCP2 cells. Then, we assayed this dynamically generated CM to modulate osteoclastogenesis.

We challenged this optimized preclinical model with Gef and bone-targeted therapies and Eve to evaluate the contribution of the EGFR pathway to the interaction between MSC and SCP2 cells, as well as to the osteoclastogenic effect of the CM.

Lu et al. showed that Gef significantly reduced the development of metastasis after intracardic or intratibial inoculation of mice with SCP20 cells, a highly bone-metastatic clone of the MDA-MB-231 cells [19]. Normanno et al. extensively investigated the contribution of the EGFR pathway to the crosstalk of different stromal cell populations within the bone marrow, and of stromal cells with cancer cells. They showed relevant cytological effects of Gef in the setting assayed, including the direct cytotoxic effect of the drug and the inhibition of osteoclastogenesis [17,46,49].

Our results indicated that MSC induced the upregulation of RANK in SCP2 and, to a lesser extent, of the EGFR pathway, suggesting that the crosstalk with MSC promoted the stimulation of these two signaling pathways. Considering that RANK, as previously discussed, is related to osteomimicry, this remarkable observation supports that our experimental approach is informative of the molecular interactions occurring in vivo. On the other hand, EGFR is the target of Gef: its increase after COCO with bone cells endorses the biological rationale to use this drug for inhibiting cancer cells in the bone microenvironment.

Treatment of MSC-SCP2 COCO with Gef only partially altered this upregulation. This finding should undergo further investigation as recent studies have evidenced the interaction between these two signaling pathways, for example in bone metastasis from soft tissue sarcomas [50]. Interestingly, the CM from MSC-SCP2 COCO seemed to induce osteoclastogenesis more efficiently than the CM from either MSC or SCP2 monocultures. The treatment of MSC-SCP2 COCO with Gef, although not affecting the number of induced osteoclasts, significantly reduced their relative surface area, thus highlighting an interfering effect of this drug on the osteoclastogenesis process.

When the osteoclasts were treated with BTT and Eve, we observed that all drugs showed an inhibition effect on osteoclastogenesis. Zol and Eve performed the strongest inhibition on the development of mature osteoclasts in the MSC-sample, cultured singly or co-cultured with SCP2.

## 4. Materials and Methods

### 4.1. Cell Cultures and Reagents

The SCP2 cell line was kindly provided by Yibin Kang laboratory, where these cells had been initially isolated, as an osteotropic clone of the BC cell line MDA-MB-231 [38]. Human MSC where purchased from Lonza (Lonza, Basel, Switzerland). Cells were cultured as a monolayer in 75 cm^2^ flasks at 37 °C in a 5% CO_2_ atmosphere in DMEM medium (Euroclone, Milan, Italy) supplemented with 10% fetal bovine serum (Euroclone, Milan, Italy), 1% penicillin/streptomycin (PAA, Piscataway, NJ, USA) and 1% glutamine (PAA), referred as “complete medium”.

### 4.2 Drug Sensitivity Assay

To assess SCP2 sensitivity to Gef, the 3-(4,5-dimethylthiazol-2-yl)-2,5-diphenyltetrazolium bromide (MTT) assay was performed (Sigma-Aldrich, Steinheim, Germany). Five thousand SCP2 cells were plated in a 96-well plate and treated for 24 h with Gef at the following concentrations: 0.0625, 0.125, 0.25, 0.5 and 1 µg/mL. After treatment, medium was discarded and cells were cultured with fresh DMEM for further 24 h, for washing out the drug. Then, a stock solution of MTT (5 mg/mL) was diluted 1:10 in fresh DMEM and 100 µL were dispensed in each well. Cells were incubated for 2 h at 37 °C in the dark. After incubation, the MTT solution was discarded and cells were incubated with 100 µL of an HCl isopropanol solution in order to solubilize formazan crystals. Absorbance was determined by spectrophotometric measurement at 550 nm.

### 4.3. MSC-SCP2 COCO Assay and Generation of CM

For COCO assays, 2 × 10^5^ MSC were plated in 6-well plates and, separately, 1 × 10^5^ SCP2 cells were plated in a 24 mm diameter transwell insert with 0.4 µm pores (Corning Ltd., Flintshire, UK). Cells were then allowed to adhere to their supports and, after 24 h, SCP2-seeded inserts were placed over the MSC cultures to start COCO. One SCP2-seeded insert was harvested for gene expression analysis at baseline. Cells were co-cultured in 4 mL complete DMEM for 24 h, then medium from COCOs was harvested to produce the Early-CM and replaced with 4 mL fresh complete DMEM, either or not supplemented with 1 µg/mL Gef. After 24 h of refreshment, media were completely discarded and cells were cultured in fresh complete DMEM, for washing out the drug. After further 24 h, COCOs were stopped to harvest the Late-CM from the cells not treated with Gef, and the Gef-CM from the cells treated with Gef. CM were harvested also from MSC and SCP2 cultured separately. CM were collected, centrifuged for 5 min at 1200 rpm and immediately stored at −20 °C. SCP2 and MSC were harvested for RNA and protein extraction, respectively.

### 4.4. Generation of Pre-Osteoclasts

Human pre-osteoclasts were generated from PBMC, following a previously established protocol with some modifications [12]. Briefly, PBMC of healthy donors who had given their written informed consent were separated by Ficoll density centrifugation (Lymphosep, Biowest, Nuaillé, France), counted and seeded in αMEM medium (Lonza) supplemented with 10% FBS, 1% penicillin/streptomycin, 1% glutamine (referred to as complete αMEM), in 24-well plates at a density of 750,000 cells/cm^2^. After 2–3 h the medium was replaced with pre-osteoclast medium (complete αMEM supplemented with 20 ng/mL recombinant human M-CSF, Peprotech, Rocky Hill, NJ, USA). Pre-osteoclasts were cultured in pre-osteoclast medium, with medium refreshment every 3 days until cultures had reached about 80% confluency.

### 4.5. Osteoclastogenesis Assay with CM and Drugs

Pre-osteoclasts were cultured either in DM (complete αMEM supplemented with 20 ng/mL M-CSF and 20 ng/mL RANKL), or in pre-osteoclast medium (as a negative control for osteoclastogenesis) or in pre-osteoclast medium supplemented with 20% of CM collected in the COCO assay. Culture conditions for osteoclastogenesis assay are summarized in Table 1.

After 14 days of the beginning of the experiment, cells were fixed by incubation in 3.7% PBS buffered formaldehyde (Polyscience, Niles, IL, USA) for 10 min at room temperature and then stained for tartrate-resistant acid phosphatase (TRAP kit, Sigma-Aldrich, Steinheim, Germany). Nuclei were counterstained with hematoxylin (TRAP kit). Multinucleated (>4 nuclei), TRAP-positive cells with at least 3 nuclei were marked as osteoclast cell-like cells. To measure the extent of osteoclastogenesis in the different experimental conditions, we measured the osteoclast surface area. Three pictures per well per culture condition were taken with AxioVision software (ZEISS, Oberkochen, Germany) and the surface area of osteoclasts was measured with ImageJ software (http://imagej.nih.gov/ij, NIH, Bethesda, MD, USA). The resulting total area was then divided by the number of measured osteoclasts, to obtain the average value. Experiments were done in triplicate.

Osteoclastogenesis assay was performed also in presence of drugs. Eve 0.1 µg/mL was added (Afinitor^®^, Novartis, East Hanover, NJ, USA) on day 3, and Zol 10 µmol (Zometa^©^, Novartis, East Hanover, NJ, USA) and Den (5 µg/mL) (XGEVA^®^, Thousand Oaks, CA, USA) on day 7. Each drug was added for 72 h. For each condition the CM of MSC, SCP2 and the COCO treated with and without Gef were added.

### 4.6. Western Blot

For phosphorylated-EGFR (pEGFR) evaluation in MSC and SCP2, proteins were isolated by direct cell lysis with a lysis buffer composed of 50 mM Tris-HCl (pH 8), 150 mM NaCl, 1% Triton X-100 and 0.1% SDS, supplemented with 1 mM phenylmethylsulfonyl fluoride and 1:100 protease inhibitors (Sigma-Aldrich, Milan, Italy). For pEGFR positive controls, MSC and SCP2 cultures were treated with 50 ng/mL human recombinant EGF (Merck Millipore, Billerica, MA, USA) 15 min before cell lysis. The protein content was quantified using BCA protein assay kit (Thermo Fisher Scientific, Waltman, MA, USA). An equal amount of protein was separated from each sample on Bolt 4–12% Bis-Tris Plus 10 well (Novex, Life Technologies, Carlsbad, CA, USA) and transferred to Mini Format, 0.2 µm PVDF (Biorad, Hercules, CA, USA). The membranes were blocked for 2 h in 5% non-fat dry milk PBS with 0.1% Tween 20 (Sigma-Aldrich, Milan, Italy) at room temperature and incubated overnight at 4 °C with primary antibody. After washing, the membranes were incubated for 1 h at room temperature with goat anti-mouse IgG-HRP (1:5000, Santa Cruz Biotechnology, Dallas, TX, USA) and goat anti-rabbit IgG-HRP (1:5000, Santa Cruz Biotechnology) for anti-vinculin, and anti-pEGFR pretreated membranes, respectively. The acquisition was performed after 5 min of treatment with Clarity^TM^ (Western ECL Substrate, Hercules, CA, USA). The following primary antibodies were used: anti-pEGFR (Tyr1173) (1:500, Upstate Cell Signaling Solution, Lake Placid, NY, USA), anti-vinculin (1:1000, Thermo Fisher Scientific).

### 4.7. RNA Extraction and Real-time Quantitative PCR (qPCR)

SCP2 and osteoclast cultures were directly lysed with 800 μL TRIzol^®^ reagent (Life Technologies). RNA was then extracted according to the manufacturer’s protocol, resuspended in molecular-grade bidistilled water and quantified with Nanodrop-1000 (Thermo Scientific). Five hundred ng of RNA for SCP2 and 250 ng of osteoclasts were then used for RT-PCR using iScript™ cDNA Synthesis Kit (Biorad, Hercules, CA, USA) according to the manufacturer’s protocol. For qPCR, either SYBR^®^ green or TaqMan^®^ chemistry was used, according to the specific target gene assay (Table 2).

For SYBR^®^ assays, SYBR^®^ Select Master Mix (Life Technologies) was used with the following cycling conditions: 5 min at 50 °C and 5 min at 95 °C (hold), followed by 15 s at 95 °C and 60 s at 60 °C for 40 cycles, followed by melting curve stage. For TaqMan^®^ assays, TaqMan^®^ Universal PCR Master mix (Life Technologies) was used, with the same thermal profile as for intercalating dyes assays, excluding the melting curve stage. Gene expression was quantified by the Δ–Δ *C*_t_ method, normalizing samples first on the housekeeping genes *actb* and *hprt*, and then on the baseline reference samples.

### 4.8. Satistical Analysis

Each experiment was performed in triplicate. Data are presented as mean ± SD. Differences were assessed by a two-tailed Student’s *t*-test and accepted as significant at *p* < 0.05.

## 5. Conclusions

We developed a fully human COCO system of BC cells and bone progenitor cells resembling some of the molecular interactions observed in vivo. We observed that MSC interaction with cancer cells induced molecular changes in the RANK pathway necessary for osteoclastogenesis and key to the formation of bone metastasis and the EGFR pathway. Our model also confirmed that the crosstalk between cancer and bone cells is crucial for bone metastasis.

The observation of the EGFR upregulation supports our idea of challenging the model with a TKI drug, as the EGFR inhibition caused a fault in the osteoclastogenesis process. This effect was enhanced by the osteoclast treatment with either Eve or Zol. These results open the way for further investigation on the combination of conventional therapy with EGFR-targeting drugs in patients with bone metastasis.

## Figures and Tables

**Table 1:** Assay condition of the osteoclastogenesis assay.

Differentiation Medium	Pre-Osteoclast Medium	Pre-Osteoclast Medium		
		supplemented with Early-CM from:	supplemented with Late-CM from:	supplemented with Gef-CM from:
Alone (positive control)	Alone (negative control)	MSC culture	MSC culture	MSC culture
		SCP2 culture	SCP2 culture	SCP2 culture
		COCO	COCO	COCOC

**Table 2:** Primers and probes.

**SYBR™ Green Assays**			
Gene symbol	Forward primer (5’–3’)		Reverse primer (5’–3’)
*ACTB*	GCACAGAGCCTCGCCTT		CCTTGCACATGCCGGAG
*HPRT1*	AGACTTTGCTTTCCTTGGTCAGG		GTCTGGCTTATATCCAACACTTCG
*SPP1*	AGATGGGTCAGGGTTTAGCC		CATCACCTGTGCCATACCAG
*GJA1 (CX43)*	TCTGAGTGCCTGAACTTGC		ACTGACAGCCACACCTTCC
*ANGPT1*	CCGACTTCATGTTTTCCACA		ACCGGATTTCTCTTCCCAGA
*JDP2*	CTTCTTCTTGTTCCGGCATC		CTTCCTGGAGGTGAAACTGG
*CTSK*	GCCAGACAACAGATTTCCATC		CAGAGCAAAGCTCACCAGAG
**TaqMan^®^ Assays**			
Gene symbol		Assay identification number	
*ACTB*		Hs99999903_m1	
*HPRT1*		Hs02800695_m1	
*EGFR*		Hs01076078_m1	
*TNFRSF11A (RANK)*		Hs00921372_m1	
*TFF1*		Hs00907239_m1	

## Abbreviations

COCO	MSC-SCP2 co-culture
CM	conditioned medium
DM	differentiation medium
Gef	gefitinib
EGFR	epidermal growth factor receptor
Eve	everolimus
MSC	mesenchymal stromal cells
GF	growth factors
M-CSF	macrophage colony-stimulating factor
MTT	(4,5-dimethylthiazol-2-yl)-2,5-diphenyltetrazolium bromide
NSCLC	non-small cell lung cancer
PBMC	human peripheral blood mononuclear cells
Den	denosumab
RANKL	receptor activator of nuclear factor kB ligand
TKI	tyrosine kinase inhibitor
TRAP	tartrate-resistant acid phosphatase
WB	western blot
